# Identifying Influential Nodes Based on Evidence Theory in Complex Network

**DOI:** 10.3390/e27040406

**Published:** 2025-04-10

**Authors:** Fu Tan, Xiaolong Chen, Rui Chen, Ruijie Wang, Chi Huang, Shimin Cai

**Affiliations:** 1 School of Business Administration, Southwestern University of Finance and Economics, Chengdu 611130, China; ftan5967@gmail.com; 2 School of Computing and Artificial Intelligence, Southwestern University of Finance and Economics, Chengdu 611130, China; chenxiaolong@swufe.edu.cn (X.C.); chenrui062@gmail.com (R.C.); huangchi@swufe.edu.cn (C.H.); 3 Engineering Research Center of Intelligent Finance, Ministry of Education, Chengdu 611130, China; 4 School of Mathematics, Aba Teachers College, Wenchuan 623002, China; 5 Big Data Research Center, University of Electronic Science and Technology of China, Chengdu 611731, China; shimin.cai81@gmail.com

**Keywords:** complex network, influential node identification, multi-attribute features, Dempster–Shafer evidence theory, visibility graph algorithm

## Abstract

Influential node identification is an important and hot topic in the field of complex network science. Classical algorithms for identifying influential nodes are typically based on a single attribute of nodes or the simple fusion of a few attributes. However, these methods perform poorly in real networks with high complexity and diversity. To address this issue, a new method based on the Dempster–Shafer (DS) evidence theory is proposed in this paper, which improves the efficiency of identifying influential nodes through the following three aspects. Firstly, Dempster–Shafer evidence theory quantifies uncertainty through its basic belief assignment function and combines evidence from different information sources, enabling it to effectively handle uncertainty. Secondly, Dempster–Shafer evidence theory processes conflicting evidence using Dempster’s rule of combination, enhancing the reliability of decision-making. Lastly, in complex networks, information may come from multiple dimensions, and the Dempster–Shafer theory can effectively integrate this multidimensional information. To verify the effectiveness of the proposed method, extensive experiments are conducted on real-world complex networks. The results show that, compared to the other algorithms, attacking the influential nodes identified by the DS method is more likely to lead to the disintegration of the network, which indicates that the DS method is more effective for identifying the key nodes in the network. To further validate the reliability of the proposed algorithm, we use the visibility graph algorithm to convert the GBP futures time series into a complex network and then rank the nodes in the network using the DS method. The results show that the top-ranked nodes correspond to the peaks and troughs of the time series, which represents the key turning points in price changes. By conducting an in-depth analysis, investors can uncover major events that influence price trends, once again confirming the effectiveness of the algorithm.

## 1. Introduction

Due to the powerful representation ability of complex networks, they are widely used to explore the properties of the real systems [1,2,3], including disease transmission [4,5], information diffusion [6], and traffic coordination [7,8]. However, the structural properties of real-world networks are often determined by a small number of key nodes. For example, in terms of degree distribution, hub nodes play a crucial role in establishing the scale-free nature of networks [9]. In network connectivity, a few important nodes act as bridges that link different communities or subnetworks [10]. Moreover, in power networks or financial networks, some nodes are important for enhancing network robustness against targeted or random attacks [11,12]. For example, in financial networks, critical nodes often refer to financial institutions with large asset sizes and frequent transactions; for instance, large commercial banks or state-owned banks, due to their broad influence in the market, may become potential sources of risk. If these banks encounter financial crisis, a ripple effect could cause other financial institutions to face liquidity problems, thereby triggering systemic risk. Identifying influential nodes can help us to understand which institutions have the greatest impact on the stability of the entire financial system, and thus propose targeted defense measures [13]. In terms of propagation dynamics, key nodes are often central in the dissemination of information, resources, or pathogens. For instance, during the COVID-19 pandemic [14], a small number of individuals could infect a large number of those susceptible to the disease; these individuals are known as super spreaders. Isolating these nodes can effectively curb the spread of the virus. Sartori et al. [15] compared the effects of seven node vaccination strategies in twelve real-world complex networks. The node vaccination strategies were modeled as a form of node removal in the network. Experiments were conducted using both non-adaptive and semi-adaptive methods to quantify the effectiveness of each strategy. The results showed that the optimal strategy varies with the method used and is influenced by the availability of vaccines. A partial recalculation of node centrality improved the effectiveness of the strategies by up to 80%. Saunders et al. [16] proposed a network model to study the effects of different immunity and vaccination scenarios on the transmission of COVID-19. They found that the average immunity duration after infection is a key parameter. Additionally, they simulated various vaccination strategies, demonstrating that prioritizing vaccinations for highly connected individuals is the fastest strategy for controlling the pandemic.

Thus, it is evident that mining and identifying key nodes or influential nodes in networks is a very important and practically significant research topic in network science, and has consequently attracted the participation of many researchers [17].

Currently, a large number of algorithms have been proposed to identify influential nodes in complex networks. The methods of these algorithms are mainly based on low-order and high-order networks, respectively. The low-order network-based methods use the structural properties of the network to evaluate the importance of nodes by calculating their degree centrality [18], betweenness centrality [19], closeness centrality [20], and other classic centrality indicators [21,22,23,24]. These works instigated research on influential node identification. Since then, algorithms for identifying influential nodes have begun to emerge. For example, Zhang et al. [25] introduced a deep learning-based method which combines convolutional neural networks and graph neural networks. The simulation results show that this method outperforms traditional methods. Kou et al. [26] proposed a graph multi-head attention regression model that aggregates the features of neighbors; it can effectively identify key nodes in social networks. Li et al. [27] analyzed the propagation ability of central nodes by assigning different weights to each type of neighbor and aggregating their contributions. Wang et al. [28] conducted a study on identifying the influential nodes based on the centrality metrics. These methods have laid the foundation for further research into the properties of complex networks.

However, further research indicates that relationships between individuals are not only simple pairwise interactions but also complex triadic or polyadic relationships. For example, in social networks, interactions among three or more people are more common than pairwise interactions [29,30]. Higher-order networks provide tools to describe such relationships, allowing us to model the complex relationships in reality more accurately [31]. Therefore, identifying important nodes in higher-order networks has become another hot topic of discussion. In hypergraphs, Kapoor et al. [32] proposed that the degree centrality of a node can be defined based on its adjacent nodes, where two nodes are considered adjacent if they belong to the same hyperedge. Kovalenko et al. [33] presented hypergraph vector centrality, which differs from traditional graph centrality. Hypergraph centrality takes into account the hyperedges in the hypergraph structure. Mancastroppa et al. [34] proposed the concept of a hypercore number which reflects the closeness of the node’s connections. Li et al. [35] defined the propagation influence of nodes based on network structure and propagation processes. Serrano et al. [36] put forward the closeness centrality, which is defined as the reciprocal of the path distance from a node to all other nodes. However, the above algorithms mostly focus on the structural characteristics of nodes at a single scale, while neglecting the multi-attribute features of the nodes. In fact, nodes in the network may exhibit different characteristics at different scales, and single-scale analysis may not fully identify key nodes. Therefore, designing a more comprehensive and integrated method is the next important task.

Thus, scholars have proposed many algorithms that integrate multiple attributes. Xu et al. [37] used a graph learning framework to integrate the features of the road networks. Lei et al. [38] studied the problem of identifying key nodes in undirected networks, using Taselli entropy to integrate the local and global attributes of nodes. Lee et al. [39] proposed a general iterative framework which integrated various structural information. The results showed that this method can effectively identified super spreaders in complex networks. Shang et al. [40] introduced an method which is based on edge weight updates; it incorporated some dynamic information to ensure the accuracy of the results. Wei et al. [41] integrated multiple indicators to measure node centrality in order to construct disease transmission models and targeted immunization strategies.

However, influential node identification often faces three main challenges. First, the evaluation of node importance is often accompanied by uncertainty, such as incomplete data and noise interference. Second, different metrics may sometimes yield conflicting results. For example, a node may have a high degree centrality but a low betweenness centrality, resulting in seemingly contradictory outcomes. Additionally, nodes may have different degrees of importance at different levels in social networks, and this makes it difficult to identify the truly critical nodes in the overall network structure. These issues can affect the accuracy of node identification, and it is clear that the previously mentioned algorithms cannot fully resolve these problems.

The Dempster–Shafer evidence theory is a method for handling uncertain reasoning and information fusion; it is widely applied in many fields [42]. First, the theory quantifies uncertainty through its basic belief assignment function and combines evidence from different information sources, allowing it to effectively handle uncertainty [43]. Second, Dempster–Shafer evidence theory processes conflicting evidence through Dempster’s rule of combination, enabling the reasonable fusion of conflicting information. This avoids discarding potentially useful indicators; thereby, it improves the robustness of key node identification. Lastly, Dempster–Shafer evidence theory can also be used to integrate evidence from different layers. For example, in multilayer social networks, the importance of nodes may vary across layers yet remain interrelated. The Dempster–Shafer evidence theory can synthesize information from different layers, helping to identify truly critical nodes within the overall network structure.

Existing studies have utilized the D-S evidence theory to identify key nodes and have proposed different fusion strategies. Wei et al. [44] integrated node degree and weight to assess influence, making their method suitable for weighted networks but without considering multiple centrality measures. Li et al. [45] investigated Networks of Networks (NON) and applied D-S evidence theory to fuse cross-layer node influence, primarily relying on closeness centrality and distance matrices. Mo et al. [46] proposed the MeC method, which integrates multiple centrality measures but is only applicable to undirected and unweighted networks. Therefore, it is necessary to propose a more comprehensive evaluation method and optimize the weighted fusion strategy based on D-S evidence theory to improve the accuracy and applicability of key node identification in single-layer complex networks.

Based on the above analysis, a comprehensive evaluation method based on Dempster–Shafer evidence theory (DS) is proposed in this paper, which comprehensively considers degree centrality (DC), betweenness centrality (BC), closeness centrality (CC), harmonic closeness centrality (HCC), PageRank (PR), and eigenvector centrality (EC). This method builds a more complex and precise model through data fusion. To verify the effectiveness of the DS algorithm, we conduct robustness experiments on real complex networks. The results show that, compared to other algorithms based on six single metrics, the DS algorithm identifies influential nodes more accurately. Furthermore, to validate the applicability of the algorithm, we used it in financial networks. The analysis revealed that the algorithm could effectively identify significant events in the financial network, once again confirming its effectiveness. Specifically, we have noted a recent study [47] that is conceptually similar to our approach. However, that work only employs degree, betweenness, closeness, and eigenvector centrality. In contrast, our method introduces DS evidence theory-based fusion and additionally incorporates harmonic closeness centrality and PageRank, making power user identification more robust and accurate. Furthermore, our approach is validated using real-world datasets, whereas the aforementioned study relies solely on Threads data, limiting its generalizability. By leveraging multiple centrality measures, our fusion strategy mitigates biases from individual metrics, ensuring superior accuracy, robustness, and applicability, making it more suitable for influence analysis in complex networks.

**Figure 1:**
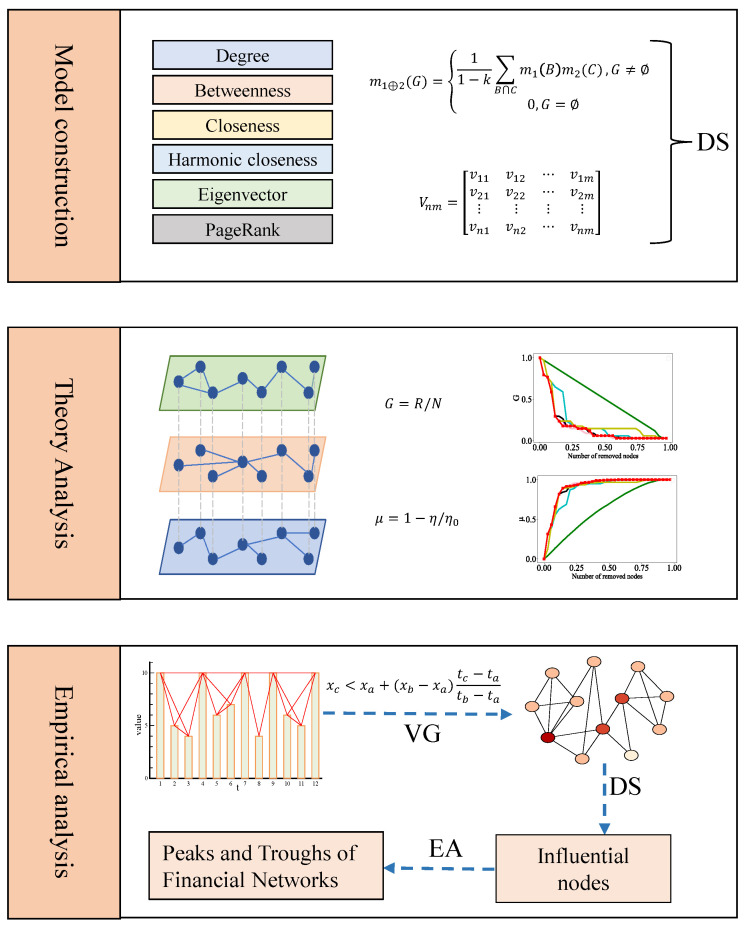
The framework diagram of this paper. In the model construction section, the acronym DS represents DS evidence theory; in the theoretical analysis section, G on the *Y*-axis denotes the largest connected subgraph, and μ on the *Y*-axis represents network decomposition efficiency; in the empirical analysis section, the acronym VG represents visibility graph, EA refers to empirical analysis, t denotes time, and value represents the corresponding numerical value at that time.

The remainder of this paper is organized as follows. In Section 2, the algorithms and models required for futures trading network analysis are described in detail. Section 3 presents the evidence theory and algorithm framework used in this paper. Detailed analysis results and discussions are provided in Section 4. Finally, Section 5 offers the corresponding conclusions and discussions; the framework diagram of this paper is shown as in Figure 1.

## 2. Preliminaries

To facilitate an understanding of the content of this article, we sequentially introduce a visibility graph algorithm (VG), centrality indicators of complex networks and an evidence theory algorithm.

### 2.1. Visibility Graph Algorithm

**Figure 2:**
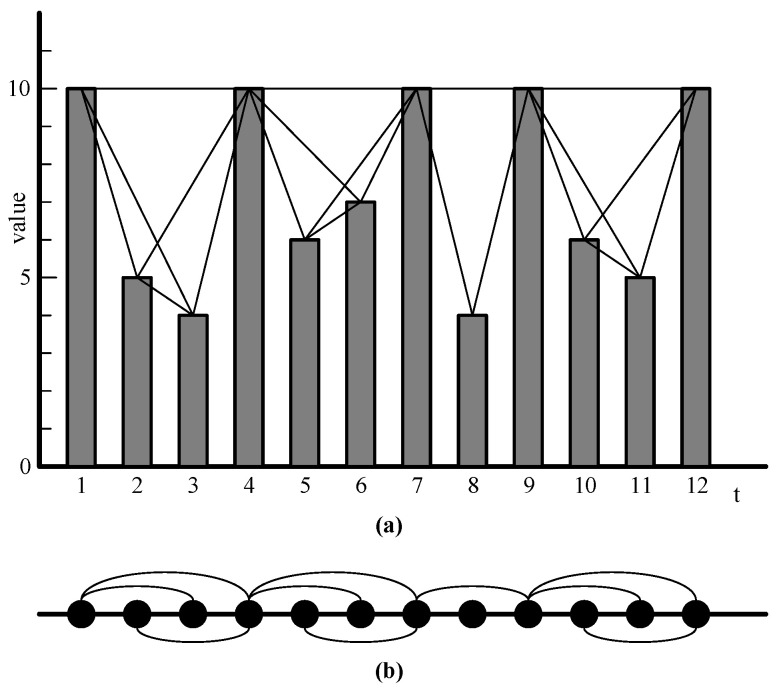
The construction of the complex network. Subgraph (**a**) illustrates the time series, where the X-axis represents time t, the Y-axis represents the corresponding value at time t, and the edges indicate the connections between these values. Subgraph (**b**) represents the abstracted network derived from Subgraph (**a**).

Time series mapping is a method of transforming time series data into complex networks, which can reveal hidden relationships, patterns, and dynamic features in the data. In 2008, Lacasa et al. [48] first proposed the visibility graph (VG) algorithm. Every node in the network corresponds to every time point in every piece of data in the discrete time series. As shown in Figure 2a, the 12 boxes represent the 12 data of the time series in sequence, and the height of the boxes represents the size of the data. If the tops of the two boxes are visible to each other, a straight line can be used to connect the two tops. Figure 2b shows a network generated by this method. The visibility criteria are as follows:

Given a time series $T=\{t_{1},t_{2},\cdots ,t_{n}\}$, the observed value for this variable is $X=\{x_{1},x_{2},\cdots ,x_{n}\}$; if the two points are visible, then for any point $\left(t_{a},x_{a}\right)$ and $\left(t_{b},x_{b}\right)$ where $t_{a}<t_{c}<t_{b}$, which satisfies the following formula:

(1) $$x_{c}<x_{a}+\left(x_{b}-x_{a}\right)\frac{t_{c}-t_{a}}{t_{b}-t_{a}}$$

### 2.2. Description of Centrality Indicators

A complex network is a graph composed of edges and nodes, usually represented by G=(V,E), where $V=(v_{1},v_{2},v_{3},\cdots ,v_{n})$ is the node set of the network, representing the individuals in the complex system. $E=(e_{1},e_{2},e_{3},\cdots ,e_{m})$ is the edge set of the network, representing the interrelationships between individuals.

In an undirected network, the degree centrality (DC) of a node refers to the number of edges directly connected to this node [18], which is denoted as $DC_{i}$. The formula is expressed as follows:

(2) $$DC_{i}=\sum_{j}e_{ij},$$

where $e_{ij}$ represents the edge connecting nodes i and j.

In complex network analysis, the closeness centrality (CC) measures the separation between nodes, which calculates the average distance between those nodes [20]. For an undirected graph, the closeness centrality $CC_{i}$ of node *i* can be expressed as the reciprocal of the average shortest path length from the node *i* to other nodes. The formula is as follows:

(3) $$CC_{i}=\frac{1}{\frac{1}{n-1}\sum_{j\neq i}d_{ij}},$$

where *n* is the number of nodes in the network and $d_{ij}$ is the shortest path length from node *i* to node *j*.

Harmonic closeness centrality (HCC) is an indicator used to measure the proximity of nodes in a network [21]. Unlike the general closeness centrality, the calculation of HCC considers the harmonic average of the shortest path length from a node to other nodes. The formula for calculating the HCC is as follows:

(4) $$HCC_{i}=\frac{n-1}{\sum_{j\neq i}\frac{1}{d_{ij}}},$$

where *n* is the number of nodes in the network and $d_{ij}$ is the length of the shortest path from node *i* to node *j*.

The betweenness centrality is defined as the total number of shortest path passing through the node [19]. The calculation formula is as follows:

(5) $$BC_{i}=\sum_{s\neq v\neq t}\frac{\sigma_{st}\left(i\right)}{\sigma_{st}},$$

where $BC_{i}$ is the betweenness centrality of node *i*, $\sigma_{st}$ is the the number of shortest path from node *s* to node *t*, and $\sigma_{st}\left(i\right)$ is the number of shortest path through node *i*.

PageRank (PR) developed by Larry Page and Sergey Brin [49]; the purpose of this algorithm was to measure the importance of web pages in search engine results. The mathematical expression of PR can be expressed in the following form:

(6) $$PR_{i}\left(t\right)=\sum_{j=1}^{n}a_{ij}\frac{PR_{j}\left(t-1\right)}{k_{j}^{out}},$$

where $PR_{i}\left(t\right)$ is the PR value of node *i* and $k_{j}^{out}$ is the out degree of node *j*. The algorithm iterates until the PR value reaches a stable state.

The eigenvector centrality (EC) depends on the centrality of the nodes that they are connected to [22]. The calculation of EC involves the adjacency matrix of the network and its mathematical expression is as follows:

(7) $$EC_{i}=\frac{1}{\lambda }\sum_{j=1}^{n}A_{ij}\Psi_{i},$$

where $\Psi_{i}$ is the eigenvector centrality of node *i*, the λ is its maximum eigenvalue, $A_{ij}$ is the adjacency matrix of the network, and *n* is the number of nodes in the network.

### 2.3. The Dempster–Shafer Evidence Theory

Dempster–Shafer evidence theory is a mathematical framework used to handle uncertainty [42]. It is mainly used for inference in situations of uncertainty or where there is a lack of information, rather than relying on traditional probability theory. The basic idea of Dempster–Shafer theory is to assign trust to different assumptions, allowing for a more flexible modeling of uncertainty.

Assuming that there is a decision problem, the identification framework for this decision problem is $\Theta =\{\theta_{1},\theta_{2},\cdots ,\theta_{n}\}$, and all elements in this identification framework satisfy the condition of mutual exclusion. In addition, the set composed of all subsets in Θ is called the power set of Θ, represented by $2^{\Theta }$, and its form is described as follows:

(8) $$2^{\Theta }=\left\{\emptyset ,\left\{\theta_{1}\right\},\left\{\theta_{2}\right\},\cdots ,\left\{\theta_{1},\theta_{2}\right\},\cdots ,\left\{\Theta \right\}\right\}$$

The ∅ represents an empty set, and the power set contains $2^{n}$ elements. In the model defined by Shafer, the basic probability assignment (BPA) of any subset *G* on a power set is the mapping of its power set $2^{\Theta }$ to [0,1], denoted as $m:2^{\Theta }\to \left[0,1\right]$; this is the case if the following conditions are met:

(9) m(∅)=0

(10) $$\sum_{G\in 2^{\Theta }}m(G)=1$$

For $\forall G\subseteq 2^{\Theta }$ with m(G)>0, *G* is called the focal element of evidence *m*. Here, m(G) represents the BPA of subset *G*, indicating the level of evidence supporting subset *G*. With the previous definition, assuming $m_{1}\left(B\right)$ and $m_{2}\left(C\right)$ are two independent BPAs, according to the Dempster combination rule, they are fused, and the result can be described as follows:

(11) $$m_{1⊕2}\left(G\right)=\left\{\begin{array}{c} \frac{1}{1-k}\sum_{B⋂C=G}m_{1}\left(B\right)m_{2}\left(C\right),G\neq ⌀\\ 0,G=⌀ \end{array}\right.$$

where $k=\sum_{B\cap C=\emptyset }m_{1}\left(B\right)m_{2}\left(C\right)$ represents the conflict coefficient used to measure the degree of conflict between two pieces of evidence. The larger *k*, the greater the conflict between the two pieces of evidence.

## 3. The Proposed Method

The evaluation of node importance in complex networks is often accompanied by uncertainty, such as issues like incomplete data and noise interference. Additionally, in complex networks, different metrics may sometimes yield conflicting results, leading to seemingly contradictory outcomes. Furthermore, in social networks, nodes have varying levels of importance at different layers, making it challenging to identify the truly critical nodes within the entire network structure. The Dempster–Shafer evidence theory can effectively address these problems, and thus we propose a DS algorithm based on this theory. The specific operational steps are as follows:

Step 1: Use the centrality indicators of the network to obtain the multi-attribute values of the network.

Step 2: Construct an evaluation matrix $B_{nm}$ using the multiple attribute values mentioned above, where each column of the matrix represents a type of attribute value. The symbol $b_{ij}\left(i=1,2,\cdots ,n:j=1,2,\cdots ,m\right)$ represents the elements, where *n* is the number of nodes and *m* is the number of evaluation indicators.

(12) $$B_{nm}=\left[\begin{array}{cccc} b_{11} & b_{12} & \cdots & b_{1m}\\ b_{21} & b_{22} & \cdots & b_{2m}\\ \vdots & \vdots & \vdots & \vdots \\ b_{n1} & b_{n2} & \cdots & b_{nm} \end{array}\right]$$

Step 3: Normalize each column of the evaluation matrix $B_{nm}$ using the following formula:

(13) $$\nu_{ij}=\frac{b_{ij}}{\sum_{i=1}^{n}b_{ij}},j=1,2,\cdots ,m$$

We then obtain a new evaluation matrix:

(14) $$V_{nm}=\left[\begin{array}{cccc} \nu_{11} & \nu_{12} & \cdots & \nu_{1m}\\ \nu_{21} & \nu_{22} & \cdots & \nu_{2m}\\ \vdots & \vdots & \vdots & \vdots \\ \nu_{n1} & \nu_{n2} & \cdots & \nu_{nm} \end{array}\right]$$

Step 4: According to the theory of evidence, we will analogize the elements in each column of matrix $V_{nm}$ to the elements in the power set; their values are the basic probability assignment (BPA) of the corresponding elements, which is represented by $\nu_{ij}$ values as $m_{1}\left(B\right)$, $m_{2}\left(B\right)$ or other BPA values. The corresponding fusion formula is represented as follows:

(15) $$m_{1⊕2}\left(A\right)=\left\{\begin{array}{c} \frac{1}{1-k}\sum_{B⋂C=A}m_{1}\left(B\right)m_{2}\left(C\right),A\neq \emptyset \\ 0,A=\emptyset \end{array}\right.$$

Step 5: Through the method of Dempster–Shafer theory, the comprehensive evaluation values of complex network nodes can be obtained, and then the influential nodes among them can be selected. Finally, analyze the influential nodes in the network to provide recommendations for investors and entrepreneurs in stock investment. The detailed process of each step is shown in Algorithm 1.

Step 6: Use the VG algorithm to convert the time series data of futures trading into a complex network. Then, the entropy weight method is used to couple the complex networks corresponding to each time series to obtain a coupled network. Last, we perform an empirical analysis. The symbols used in this paper are listed in Table 1.
**Algorithm 1** Algorithm of DS1:**Data Collection and Processing**:2:       Obtain and preprocess complex network data.3:**Performing data conversion**:4:       Get the adjacency matrix of complex networks.5:  **Calculate the $DC_{i}$, $CC_{i}$, $HCC_{i}$, $BC_{i}$, $PR_{i}$ and $EC_{i}$ of node** *i*:6:**while** i≤n **do**7:      Calculate the six centrality values of each node in sequence.8:**end while**9:**for** 1→n **do**10:    Construct the evaluation matrix $B_{nm}$.11:    Normalize the $B_{nm}$, then we get a new matrix $V_{nm}$.12:    **if** $V_{nm}\neq 0$ **then**13:      Represent each column element of the matrix using the basic probability assignment (BPA) of evidence theory, calculate the DS value for each node.14:    **else**15:        The DS value is 0.16:        **break**17:    **end if**18:**end for**19:**repeat**20:    Perform steps 2, 3 and 4.21:**until** Traverse all nodes in the network22:The final DS valve is obtained.

Table 2 lists the time complexities of several different methods and categorizes them into three types: local, global, and hybrid, based on the network information included in the evaluation methods. Here, N represents the total number of nodes, M denotes the number of edges, and K indicates the number of iterations. It is evident that the time complexity of the DS method is not the lowest; however, its accuracy significantly surpasses that of the other methods ), and the network information contained in the nodes is much greater than that of the other methods. Therefore, the DS method exhibits certain advantages.

## 4. Experiment

### 4.1. Ranking the Influential Nodes

The node attack experiment in complex networks is an important research method for evaluating network robustness and identifying key nodes. By systematically removing nodes from the network, researchers can analyze how key attributes such as connectivity, average path length, and clustering coefficient change, thereby assessing the role that nodes play within the network. Node attack experiments are typically divided into two types: random attacks and targeted attacks. Random attacks refer to the random removal of nodes from a network. This method is primarily used to assess the overall robustness of the network, as most real-world networks exhibit high tolerance to random node failures. Targeted attacks, on the other hand, are based on certain node characteristics (such as degree centrality, betweenness centrality, or closeness centrality) to selectively remove the most important nodes, simulating the network’s vulnerability when facing targeted attacks. Targeted attacks typically weaken the network’s connectivity quickly, revealing the presence of key nodes.

This study focuses on targeted attacks. During the experiment, as key nodes are gradually removed, the network’s topology undergoes significant changes, which can lead to a rapid decline in network performance or even a collapse. Through such experiments, researchers can identify critical nodes within the network and propose more robust optimization strategies for network design, enhancing the network’s recovery ability when faced with attacks or failures. This has broad applications in fields such as social networks, communication networks, and transportation networks.

**Figure 3:**
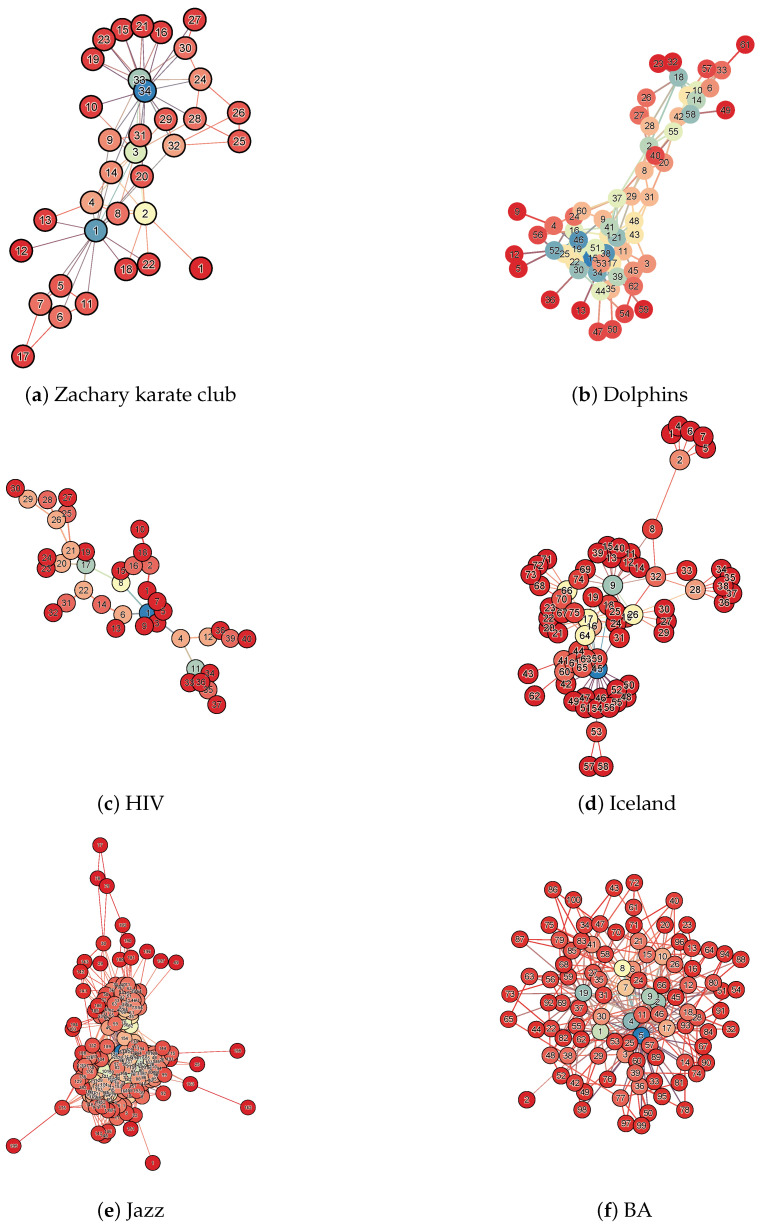
Visualization of experimental data. Subgraph (**a**) is the Zachary karate club network. Subgraph (**b**) is the Dolphins network. Subgraph (**c**) is the HIV network. Subgraph (**d**) is the Iceland network. Subgraph (**e**) is the Jazz network. Subgraph (**f**) is the BA network. In the figures, the nodes are colored in blue, light blue, orange, yellow, and red. The bluer the node, the more central its position, while the redder the node, the more peripheral its position.

In order to verify the superiority of DS method in node importance assessment, we select six real networks with different topological structures for node attack experiments. These are, namely, Zachary karate club, Dolphins, HIV, Iceland, Jazz, and Crime (http://konect.cc/networks/, accessed on 11 March 2025). The structural parameters of these networks are shown in Table 3, including their nodes (N), edges (E), diameter (DM), average shortest path (ASP), and average clustering coefficient (ACC). From these parameters, it can be seen that the size and shape of the networks are different, and they can represent different networks in the real world well. In order to visually see the topology of the network, we visualize the above network data, as shown in Figure 3. Therefore, the robustness experiments conducted on them can easily demonstrate the superiority of each centrality algorithm.

To demonstrate the effectiveness and advantages of our proposed algorithm, we make a detailed comparison between the results of this algorithm and traditional algorithms, such as DC, CC, HCC, BC, PR, and EC. Based on the ranking results of these seven algorithms, the top ranked nodes of each algorithm are removed to simulate the changes in the size of the maximum connectivity subgraph when the network is deliberately attacked; this enables us to evaluate the accuracy of each ranking algorithm. If a node is attacked according to the sorting results of a certain algorithm, the maximum connectivity subgraph size of the network decreases very quickly and this indicates that the algorithm identifies influential nodes more accurately.

In order to quantitatively analyze the changes in the size of the Largest Connected Component (LCC), the definition of the Largest Connected Component (LCC) or Giant Component [50] is as follows: In an undirected graph G=(V,E), a connected component is a subgraph in which there exists a path between any two nodes, and no node in this subgraph is connected to any node outside of it. The Largest Connected Component (LCC) refers to the connected subgraph in *G* that contains the most nodes. If *G* is a connected graph, then the LCC is the entire graph. If *G* is a disconnected graph, the LCC is the largest among all connected components.

**Figure 4:**
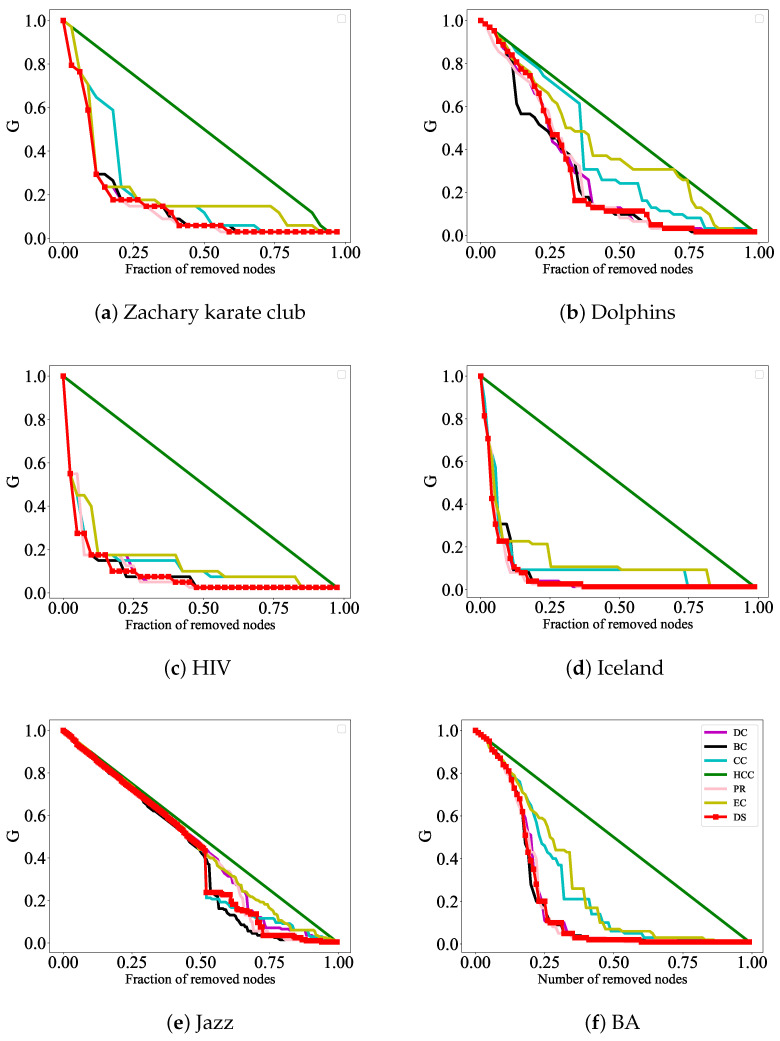
Destructive power of nodes in different networks. Key nodes identified by different methods are selected as attack targets to measure their influence. In the figure, the horizontal axis represents the number of nodes removed, and the vertical axis represents the network connectivity coefficient G=R/N. The faster the connectivity coefficient *G* decreases within the same timeframe, the greater the influence of the node.

**Figure 5:**
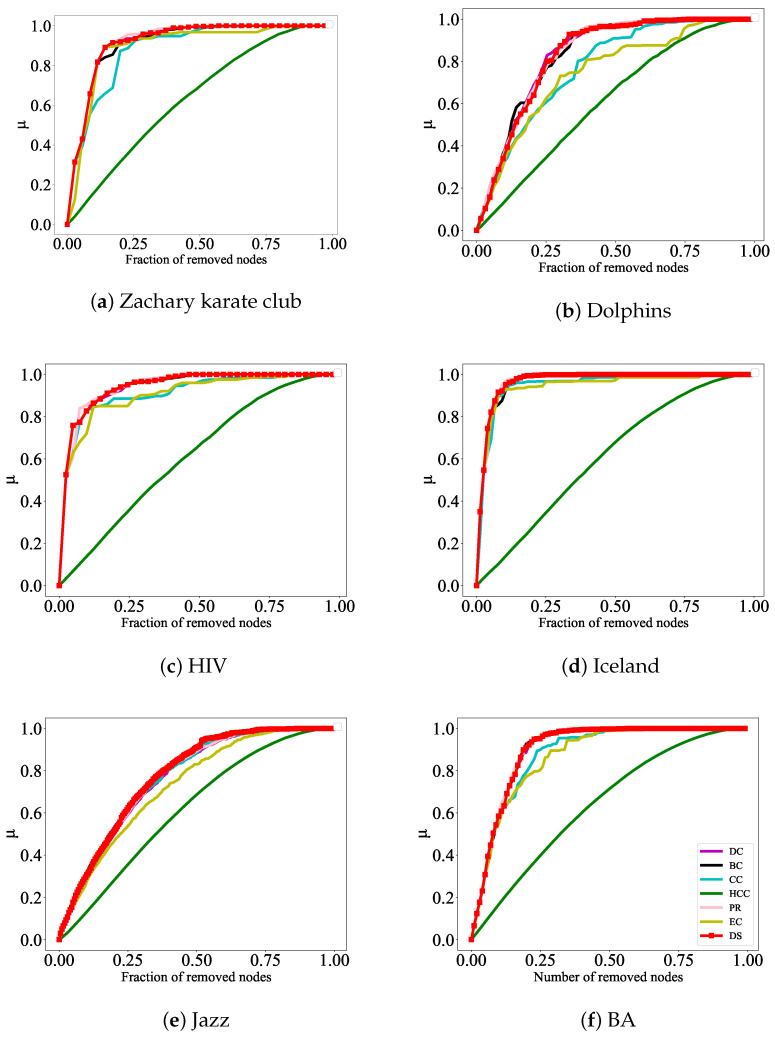
Efficiency analysis for six real networks. A certain percentage of nodes are gradually removed to measure their influence. The horizontal axis represents the number of nodes removed, and the vertical axis represents network efficiency $\mu =1-\eta /\eta_{0}$. Within the same timeframe, the faster the network efficiency μ increase, the greater influence the node has.

The formula G=R/N is used to characterize the efficiency of network decomposition, where *N* represents the number of nodes in the initial network and *R* represents the number of nodes in the maximum connectivity subgraph after removing nodes. The faster the *G* decreases, the more accurate the method identifies influential nodes. In addition, the network efficiency formula $\mu =1-\eta /\eta_{0}$ is also used to evaluate the strength of network connectivity; here, $\eta =\frac{1}{N(N-1)}\sum_{i,j\in V}\eta_{i,j}$, where $\eta_{i,j}=1/d_{ij}$. It is obvious that $d_{ij}$ represents the shortest distance between nodes. The faster the μ rises, the more accurately the method identifies influential nodes. The effect of the experiment is reflected in Figure 4 and Figure 5 below.

In Figure 4, the DS, DC, CC, HCC, BC, PR, and EC rank the nodes in six actual networks; the top ranked nodes of each algorithm are removed and the changes in the size of the network’s maximum connectivity subgraphs are observed to evaluate the accuracy of each sorting algorithm. The experimental results show that when attacking nodes according to the ranking results of the DS method, the maximum connectivity subgraph size of the network decreases the fastest, especially in Figure 4a,c,d, where the initial process of node attacks shows better attack effects than other indicators. In Figure 4b,e,f, although the initial performance of node attacks was not the best, as the experiment continued, removing the nodes ranked according to the DS method, the maximum connectivity subgraph of the network was the first to decrease to 0.

In Figure 5, the DS algorithm, along with the other algorithms, is used to rank the nodes. Based on the ranking results, the top-ranked nodes from each algorithm are removed, and the changes in the network efficiency are observed to evaluate the accuracy of each ranking algorithm. The experimental results show that when nodes are attacked based on the DS algorithm’s ranking, the network efficiency increases the fastest, especially in Figure 5c,d, where the initial phase of node attacks exhibits better performance compared to other metrics. In Figure 5b, although the initial phase of node attacks does not show the best results, as the experiment progresses, removing the nodes ranked by the DS algorithm results in the network efficiency rising to 1 the earliest. Therefore, from these two aspects, the important nodes identified by this algorithm are more accurate compared to those identified by the other six algorithms.

### 4.2. Empirical Analysis

In Section 4.1, we demonstrated the superiority of the DS algorithm from a theoretical perspective. However, the effectiveness of this algorithm in practical applications is still unknown. Therefore, we collected time series from online platforms to test whether the proposed method can identify major events in the sequence.

Sina Finance, as one of China’s leading financial portal websites, carries rich financial information and services, providing comprehensive, timely, and professional financial information and data support for investors. Its platform encompasses information from multiple fields such as stocks, funds, futures, foreign exchange, bonds, etc., providing users with comprehensive investment decision support. Therefore, we collect the trading data of GBP futures from 1 January 2020 to 1 October 2023. Then, we analyze the trend of GBP futures to provide corresponding decision-making guidance for investors. It is worth mentioning that there are no missing values in the data, which also ensures the validity of the experimental results.

**Figure 6:**
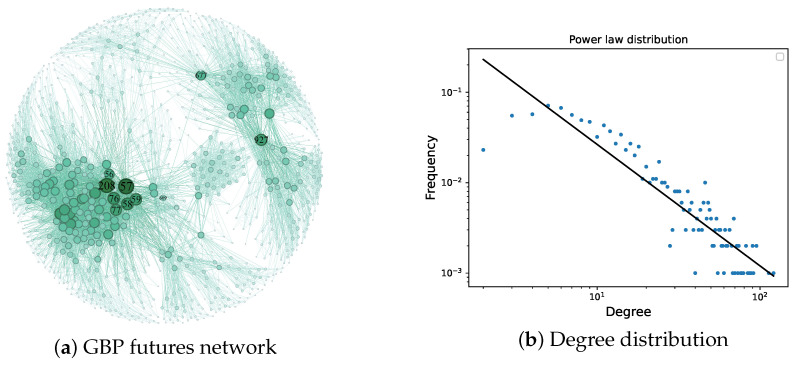
GBP futures network and its degree distribution.

The network constructed by the VG algorithm based on the above data exhibits power-law distribution characteristics, as shown in Figure 6. The power-law distribution is widely present in the real world, such as in the urban population, wealth distribution, and paper citations. Therefore, when complex networks exhibit a power-law distribution, they are more closely aligned and reflect the complexity and diversity of the real world. This distribution characteristic helps us to have a deeper understanding of and provides an explanation for various phenomena in the real world and demonstrates the rationality of the constructing method.

So, in this section, we will use the DS method to identify influential nodes in the GBP futures network, and correspond the identified influential nodes to the corresponding time points in the time series. Then, we will analyze the price characteristics of GBP futures at that time point, and based on this feature, identify important events that affect GBP futures trading on that day. Our ultimate goal is to provide relevant investment advice for futures investors. In Table 4, based on the DS algorithm, this article lists the top ten influential nodes, their corresponding times, and major events that occurred on that day.

Economic policies and political events affect futures trading by changing market expectations, capital flows, and supply–demand relationships. For example, the easing or tightening of monetary policy directly affects the cost of market funds, thereby affecting the investment activity of the futures market; the adjustment of fiscal policies affects government spending and taxation, thereby affecting the supply and demand relationship and price trends of related industries; the occurrence of political events may trigger panic in the market, leading to a decrease in investor confidence in the futures market and subsequently affecting market prices. Next, we analyze the economic policies and political events underlying the key nodes identified by the algorithm proposed in this study.

Node 10 corresponds to 9 March 2020; this day marked by a sharp plunge in the opening prices of the New York Stock Exchange (NYSE). With losses reaching 7%, it triggered the circuit breaker mechanism. The losses then temporarily narrowed after trading resumed, but all three major NYSE indices closed with declines exceeding 7%. This event had a severe negative impact on pound futures trading, which also hit its lowest level on the same day.

The node 677 represents 24 February 2021; this was the third day after the UK Prime Minister announced the lifting of COVID-19 lockdown measures. By this time, the UK was gradually emerging from the impacts of the pandemic. Barbershops, museums, libraries, and zoos began to reopen for business, and GBP futures subsequently rose. Node 609 corresponds to 31 May 2021; this day marked the start of the second phase of lifting lockdown restrictions. While GBP futures continued their upward trend, the growth was less pronounced than the previous phase, as residents no longer exhibited a surge in pent-up consumption. In terms of the GBP futures network, this reflects that node 609 has a lower importance score compared to node 677. Node 208 corresponds to 14 December 2022; this was a date where the UK’s inflation rate declined more than expected. This significantly enhanced the domestic value of the currency and increased consumer purchasing power, which further drived the rise in pound futures.

Node 77 relates to 15 June 2023, when the Federal Reserve announced a pause in interest rate hikes to help stabilize global financial markets and alleviate capital outflow pressures from emerging markets. Node 76 represents 16 June 2023, where HSBC revealed that UK market expectations were relatively optimistic; GBP futures had also risen for two consecutive days. Node 59 relates to 11 July 2023, when the UK Energy Group reached a major agreement to import more natural gas from the United States, which would strengthen energy security in the UK, and the GBP futures continued to rise. Node 58 corresponds to 12 July 2023; the Bank of England released its stress test results for the year 2022/2023, which showed that large banks in the UK have the ability to resist significant risks, stabilizing the domestic economic situation and further boosting the GBP futures. Node 57 relates to 13 July 2023, when Goldman Sachs raised its GDP growth forecast for the UK to 0.3%, greatly boosting market confidence, and the GBP futures also reached their peak since July. Node 56 corresponds to 14 July 2023, when the results of the second round of the Conservative Party leadership election in the UK were announced. Due to the current political events, the trading price of the GBP futures had fallen compared to the previous day. This pattern is reflected in the GBP futures network, where node 57 has the highest importance characterization value, followed by node 56. Node 58 holds the importance characterization value after node 56, node 59 holds the importance characterization value after node 58, node 76 holds the importance characterization value after node 59, and node 77 holds the importance characterization value among the aforementioned nodes.

**Figure 7:**
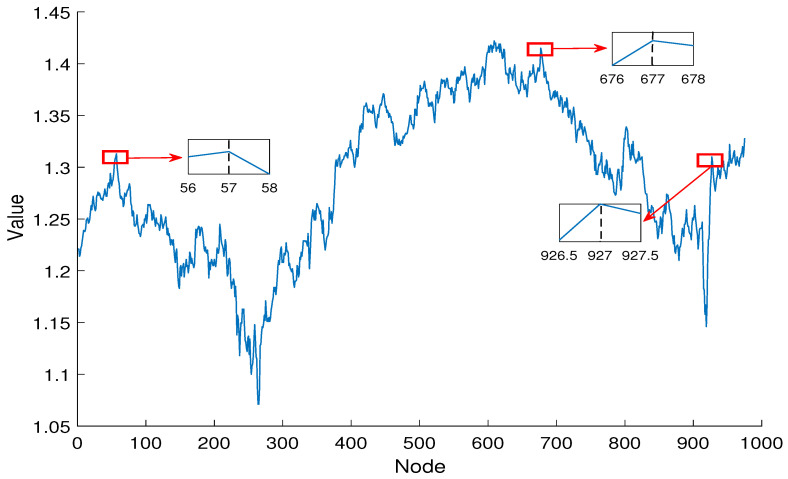
GBP futures time series.

As shown in Figure 7, to analyze the above results more intuitively, we mark the influential nodes on the the GBP futures time series. It can be observed that the influential nodes are located at the peaks and troughs or near to these. Peak and trough analysis is an important tool in financial time series for identifying trends and periodic fluctuations. Peaks and troughs reflect critical turning points in price changes, helping investors to gauge market sentiment and potential buying and selling opportunities. Through the in-depth analysis of peaks and troughs, investors can formulate more effective trading strategies, reduce risks, and seize profit opportunities. This type of analysis is applicable not only to the stock market but also to forex, commodities, and other financial markets, assisting investors in making more informed decisions.

Overall, the DS algorithm can accurately identify the key factors that affect futures trading. Investors need to closely monitor the changes in these key factors when conducting futures trading in order to develop reasonable investment strategies. At the same time, the futures market also needs to strengthen risk management and regulatory efforts to cope with potential market risks.

## 5. Conclusions

Identifying influential nodes in complex networks is a core issue in the study of complex networks. Complex networks are widely present in various practical systems, such as social networks, transportation networks, biological networks, and information networks. The influential nodes in these networks is of great significance for understanding the dynamic behavior of complex systems, improving network robustness, and optimizing resource allocation. However, traditional key node identification algorithms are typically based on a single attribute of nodes or the simple fusion of a few attributes. With further research, these identification methods have gradually revealed their limitations, as they cannot effectively handle the uncertainty, conflicts, and multidimensionality inherent in node information. To address this, this paper proposes a DS algorithm which can comprehensively consider features of nodes.

In order to compare the advantages and disadvantages of the DS algorithm with six other algorithms, we conduct resilience experiments on the Zachary karate club, Dolphins, HIV, Iceland, Jazz, and Crime networks. The results show that attacking the key nodes identified by the DS algorithm results in faster network decomposition; this indicates that the accuracy of the DS algorithm is stronger than other algorithms. Subsequently, we conduct empirical research; this experiment applies the DS algorithm to identify influential nodes in the GBP futures trading network and we find that these nodes correspond to significant events. This further demonstrates the reliability of the DS algorithm.

Based on the above findings, our work has certain practical significance. For example, in financial networks, the influential nodes are often the connecting cores of financial entities. Once these nodes are attacked, the stability of the entire financial network may be severely affected. Therefore, by enhancing the security of these critical nodes, the defense capability of financial networks can be improved to prevent systemic collapse. In addition, our work also has certain theoretical significance. The problem of identifying influential nodes prompts us to continuously explore the structural characteristics of networks. By studying the importance of nodes in different networks, complex network theory will be continuously enriched and deepened.

## Figures and Tables

**Table 1:** List of symbols and their descriptions.

Symbol	Description
$DC_{i}$	Degree of the node
$CC_{i}$	Closeness centrality of the node
$HCC_{i}$	Harmonic closeness centrality is an indicator used to measure the proximity of nodes in a network, it is unlike the closeness centrality
$BC_{i}$	Betweenness centrality of the node
$PR_{i}$	PageRank of the node
$EC_{i}$	Eigenvector centrality of the node
$DS_{i}$	DS valve of the node
Θ	$\Theta =\{\theta_{1},\theta_{2},\cdots ,\theta_{n}\}$ is the identification framework for the decision problem, and all elements in it satisfy the condition of mutual exclusion
$B_{nm}$	Valuation matrix fusing multiple attribute values
$V_{nm}$	Valuation matrix after normalizing
*G*	The size of the maximum connectivity subgraph G=R/N
μ	Network efficiency $\mu =1-\eta /\eta_{0}$

**Table 2:** Time complexity of different methods.

Method	Category	Time Complexity
DC	Local	O (N)
BC	Global	$O\left(N^{3}\right)$
CC	Global	O (NM)
HCC	Global	O (NM)
EC	Global	O (KM)
PR	Global	O (KM)
DS	Hybrid	O (NM)

**Table 3:** The topological characteristics of real networks.

Networks	Node	Edge	Diameter	Average Metrics	
				**Shortest Path**	**Clustering Coefficient**
Zachary karate club	34	78	5	2.408	0.571
Dolphins	62	159	8	3.357	0.259
HIV	40	41	10	4.473	0.042
Iceland	75	114	6	3.200	0.286
Jazz	198	2742	6	2.235	0.617
BA	100	294	4	2.499	0.231

**Table 4:** The influential nodes of GBP futures network.

Rank	Node	Time	The Important Events
1	57	13 July 2023	UK GDP will grow
2	208	14 December 2022	UK inflation will decrease
3	56	14 July 2023	Conservative Party leader election
4	677	24 February 2021	Epidemic lifting lockdown
5	58	12 July 2023	Bank stress testing
6	59	11 July 2023	Import agreement
7	76	16 June 2023	Market expectations
8	77	15 June 2023	Suspend interest rate hikes
9	609	31 May 2021	Epidemic lifting lockdown
10	927	9 March 2020	Stock market crash

## Data Availability

The data presented in this study are openly available at http://konect.cc/networks/.
